# Lower conversion rate with robotic assisted rectal resections compared with conventional laparoscopy; a national cohort study

**DOI:** 10.1007/s00464-021-08681-x

**Published:** 2021-08-18

**Authors:** Elisabeth Myrseth, Linn Såve Nymo, Petter Fosse Gjessing, Hartwig Kørner, Jan Terje Kvaløy, Stig Norderval

**Affiliations:** 1 Department of Gastrointestinal Surgery, University Hospital of North Norway, 9019 Tromsø, Norway; 2 Institute of Clinical Medicine, Faculty of Health Science, UiT, The Arctic University of Norway, 9019 Tromsø, Norway; 3 Department of Gastrointestinal Surgery, Stavanger University Hospital, 4068 Stavanger, Norway; 4 Institute of Clinical Medicine, University of Bergen, 5020 Bergen, Norway; 5 Department of Mathematics and Physics, University of Stavanger, 4036 Stavanger, Norway; 6 Department of Research, Stavanger University Hospital, 4068 Stavanger, Norway

**Keywords:** Robotic, Rectal resection, Conversion, Laparoscopy, Complications

## Abstract

**Background:**

Conversion from laparoscopic to open access colorectal surgery is associated with a poorer postoperative outcome. The aim of this study was to assess conversion rates and outcomes after standard laparoscopic rectal resection (LR) and robotic laparoscopic rectal resection (RR).

**Methods:**

A national 5-year cohort study utilizing prospectively recorded data on patients who underwent elective major laparoscopic resection for rectal cancer. Data were retrieved from the Norwegian Registry for Gastrointestinal Surgery and from the Norwegian Colorectal Cancer Registry. Primary end point was conversion rate. Secondary end points were postoperative complications within 30 days and histopathological results. Chi-square test, two-sided *T* test, and Mann–Whitney *U* test were used for univariable analyses. Both univariable and multivariable logistic regression analyses were used to analyze the relations between different predictors and outcomes, and propensity score matching was performed to address potential treatment assignment bias.

**Results:**

A total of 1284 patients were included, of whom 375 underwent RR and 909 LR. Conversion rate was 8 out of 375 (2.1%) for RR compared with 87 out of 909 (9.6%) for LR (*p* < 0.001). RR was associated with reduced risk for conversion compared with LR (aOR 0.22, 95% CI 0.10–0.46). There were no other outcome differences between RR and LR. Factors associated with increased risk for conversion were male gender, severe cardiac disease and BMI > 30. Conversion was associated with higher rates of major complications (20 out of 95 (21.2%) vs 135 out of 1189 (11.4%) *p* = 0.005), reoperations (13 out of 95 (13.7%) vs 93 out of 1189 (7.1%) *p* = 0.020), and longer hospital stay (median 8 days vs 6 days, *p* = 0.001).

**Conclusion:**

Conversion rate was lower with robotic assisted rectal resections compared with conventional laparoscopy. Conversions were associated with higher rates of postoperative complications.

Over the last 10 years, laparoscopic rectal resection has become the preferred approach in many countries [1, 2]. While several studies have shown favorable outcomes after laparoscopic surgery for colon cancer [3–7] with reduced rates of postoperative complications, 30-day mortality, and long-term results equal to open access surgery [8–10], the results after laparoscopic rectal cancer surgery have not been unambiguously positive. Although studies demonstrate similar short- and long-term results compared to open access surgery [11, 12], unfavorable histopathological outcomes with higher rates of positive circumferential resection margins, and lower rates of complete excision of mesorectum after TME have been reported [13, 14].

Due to a narrow operative field in the pelvis and limited instrument mobility, laparoscopic surgery for rectal cancer is technically demanding. Studies have shown conversion rates between 12 and 30% [15–18], and a need of about 150 operations to flatten the learning curve [19]. These disadvantages may be overcome with robotic assisted laparoscopic access which offers a three-dimensional view with a stable camera, better ergonomic conditions, enhanced dexterity, and instrument articulation. This might facilitate a more precise dissection with improved specimen quality. In particular, it may also reduce the need for conversion, which is associated with higher complication rates [3, 15, 20]. While several studies have shown lower conversion rates with robotic assisted laparoscopy compared to conventional laparoscopy [16, 21, 22], this could not be confirmed in the large randomized ROLARR trial [23].

The aim of this study was to assess conversion rates after standard laparoscopic versus robotic assisted laparoscopic resections for rectal cancer, as well as postoperative complications within the first 30 days and histopathological results in a national cohort from the Norwegian registry for gastrointestinal surgery (NoRGast) [24] supplied with data from the Norwegian Colorectal Cancer Registry [25].

## Materials and methods

### Study population

Patients who underwent elective major resection for rectal cancer from January 1st 2014 to December 31st 2018 were identified via the Norwegian Registry for Gastrointestinal Surgery (NoRGast) [24]. Due to some delay in data registry, and also to achieve at least 6 months follow-up, latest operation date for data extraction was set to December 2018. This national quality registry was established in 2014, and includes major gastrointestinal and hepatobiliary resections. All Norwegian hospitals performing cancer resections are obliged to report data to NoRGast which records variables that might affect surgical outcome, such as pre-operative weight loss, BMI, ECOG-status, known severe pulmonary and cardiac disease as well as operative technique, and short-term postoperative outcome measures including complications, reoperations, length of hospital stay, readmissions, and mortality rates. A detailed presentation of the registry has been published previously [24].

Patients were identified in the NoRGast database based on procedure codes according to the NCSP (NOMESCO Classification Of Surgical Procedures) [26] for rectal resection with formation of anastomosis (JGB00 through JGB07), rectal resection with end colostomy (codes JGB10 and JGB11), and abdominoperineal resections (codes JGB30 through JGB36). The procedure codes were combined with diagnosis code C20 for cancer < 15 cm from the anal verge assessed with rigid proctoscope according to the International Classification of Diseases version 10 (ICD-10) [27]. Some cases were registered with cancers located from 15 cm or lower measured on rigid proctoscope, but erroneously had received the ICD-10 code C19 for rectosigmoid cancer at discharge, and these were also included. Patients with tumors other than adenocarcinoma were excluded. Emergency procedures and all procedures commenced by open access, as well as transanal total mesorectal excisions (taTME) were also excluded.

Data were linked to the Norwegian Colorectal Cancer Registry [25] for information on preoperative work-up, oncologic treatment upfront surgery, histopathology of the surgical specimen, and 90 days mortality rate based on the patients’ individual social security numbers.

### Data quality

The coverage rate in NoRGast has increased during the study period from approximately 20% in 2014 to 75% in 2018 [28]. Variable completeness is 98–100%, much due to its web-based registration system. The Norwegian Colorectal Cancer Registry includes annually more than 90% of all patients surgically treated for rectal cancer [29]. However, this registry includes data from various sources, such as clinical reports on diagnosis and treatment, and histopathological reports. This results in some variations in variable completeness with missing data in up to 30% for some clinical variables, while variables from the histopathological reports have up to 90% completeness. However, as both registries overlap on a number of core variables, data linking results in an overall high degree of variable completeness. Patients with missing data in any variables included for analysis in this study were excluded, and number of missing values are documented in the attached tables. The manuscript was drafted in accordance to the STROBE guidelines for observational studies [30].

### Statistical analysis

Data were analyzed with SPSS version 26, (IBM, Armonk, New York, USA). For univariable analyses Pearson's Chi-square test was used for categorical data, and two-sided *T* test or Mann–Whitney *U* test for continuous data. Confidence interval (CI), standard deviation or inter quartile range (IQR) were calculated as appropriate. Univariable binary logistic regression was used to calculate unadjusted odds ratios (OR) for conversion rates, major complications, reoperations, 30 days mortality, and anastomotic leaks. A stepwise backward multivariable logistic regression model was used to further analyze the relations between different predictors and outcomes, and adjusted odds ratios were reported for the final fitted models. Variables with a *p* value < 0.2 in univariable analyses were included in the multivariable analyses. All significant variables were tested for two-way interaction, and significant interactions were included in further multivariable analyses. The significance level was set to *p* < 0.05.

A linear regression model was made with the continuous variable as dependent variable, RR or LR as fixed factors and hospitals performing RR as covariate.

To address potential treatment assignment bias, a propensity score matching was performed by including all available baseline variables. The matched sets were included in a new set of regression analyses. Match tolerance was set to 0.01, and sampling was done without replacement. Robotic assistance was used as group indicator, and baseline characteristics (age, gender, BMI, severe cardiac and pulmonary disease, diabetes, ASA-score, ECOG-score, and diabetes) were used as predictors.

Age was categorized into three groups (low < 65, mid 65–80, and high > 80). ASA-scores were grouped into low ASA-scores (scores 1–2) and high ASA-scores (scores 3–4). ECOG-scores were dichotomized into low ECOG-score (0–1) and high ECOG-score (2–4). Severe pulmonary disease was defined as having FEV1 < 50% or a vital capacity < 60% of predicted values. Severe cardiac disease was defined as NYHA classification 3–4, or severe arrythmia requiring mechanical support. Complications were recorded according to the Accordion grading system [31]. Major complications were defined as Accordion grade of 3 or higher. Briefly, Accordion grade 3 is defined as any percutaneous, angiographic or endoscopic intervention, Accordion 4 is defined as intervention in general anesthesia or single-organ failure, Accordion 5 is defined as intervention in general anesthesia plus single- or multi-organ failure. Accordion 6 is death within 30 days postoperatively. Anastomotic leak was defined as a leak requiring reoperation (grade C leaks) [32]. Only resections with formation of an anastomosis were included in analysis of anastomotic leak. Weight was classified by body mass index (BMI), and patients were grouped into 4 BMI-classes [33]; [< 18.5] [18.5–25] [25–30] [> 30]. Positive circumferential resection margin (positive CRM) was defined as CRM ≤ 1 mm, and positive distal resection margin (positive DRM) as DRM ≤ 1 mm.

## Results

### Patients

**Fig. 1:**
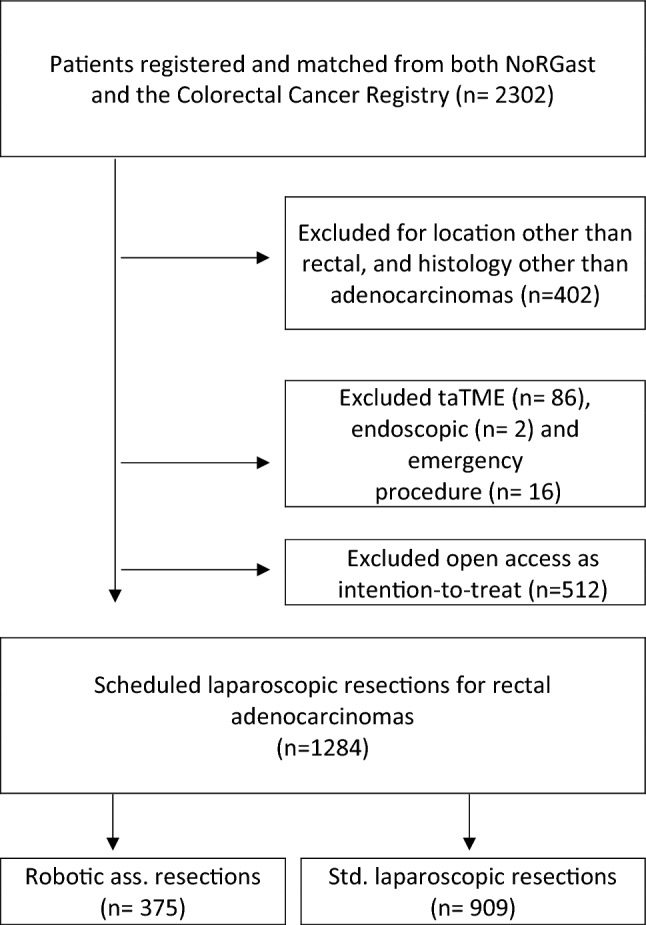
Flowchart

A total of 2302 patients were recorded in NoRGast with an NCSP procedural code for rectal resection in the study period. After excluding patients with other tumors than adenocarcinoma, those undergoing taTME, endoscopic or emergency procedures a total of 1796 patients were identified. Some 1284 had a laparoscopic procedure, of whom 909 had a conventional laparoscopic resection and 375 had a robotic assisted resection (Fig. 1). Sixteen hospitals contributed data, of which 7 performed both RR and LR. Demographical and clinical characteristics are presented in Table 1.

**Table 1:** Baseline characteristics

	Total *n* = 1284	LR *n* = 909	RR *n* = 375	*p* value*
Sex				
Male	782	551 (61)	231 (62)	0.743
Female	502	358 (39)	144 (38)	
Age, median (IQR)	69 (60–76)	69 (60–76)	69 (60–75)	0.760
BMI				
< 18.5	24	18 (2)	6 (1.7)	0.067
18.8–25	511	380 (42.4)	131 (36.1)	
25–30	496	350 (39.1)	146 (40.2)	
> 30	228	148 (16.5)	80 (22.0)	
ASA-score				
Low (1–2)	871	640 (70.4)	231 (61.6)	0.002
High (3–4)	413	269 (29.6)	144 (38.4)	
ECOG-class				
Low (0–1)	1210	854 (94.1)	356 (96.5)	0.078
High (2–4)	67	54 (5.9)	13 (3.6)	
Diabetes	134	92 (10.1)	42 (11.2)	0.565
Pulmonary disease	48	44 (4.8)	4 (1.1)	0.001
Cardiac disease	73	65 (7.2)	8 (2.1)	< 0.001
Radio(chemo)therapy	375	323 (25.5)	143 (38.1)	< 0.001
Tumor level^a^				
Low (0–5 cm)	244	159 (26.6)	85 (35.0)	0.045
Mid (5–10 cm)	332	224 (37.5)	108 (40.6)	
High (10–15 cm)	287	214 (35.8)	73 (27.3)	
Operative technique				
LAR^b^	743	552 (60.7)	191 (50.9)	0.003
APR^c^	432	280 (30.8)	152 (40.5)	
Hartmann	109	77 (8.5)	32 (8.5)	

Values in parentheses are percentages unless indicated otherwise

*LR* laparoscopic resections, *RR* robotic resections

*Chi-square analyses

^a^Cm from anal verge measured with rigid proctoscope

^b^Low anterior resection

^c^Abdominoperineal resection

### Conversion rates

The overall conversion rate was 95 out of 1284 patients (7.4%). In the RR group conversion rate was significantly lower as compared to the LR group, with 8 out of 375 (2.1%) and 87 out of 909 (9.6%), respectively (*p* < 0.001). Conversion rate for LR performed in hospitals using both operative techniques was 51 out of 464 (11.0%) compared to 36 out of 445 (8.1%) in hospitals using laparoscopic technique only (*p* = 0.137). In multivariable analyses, RR was associated with reduced risk for conversion with an aOR of 0.21 (95% CI 0.09–0.43) compared to LR. In addition, male gender (aOR 1.86, 95% CI. 1.14–3.06), BMI > 30 (aOR 2.64, 95% CI 1.51–4.61), and severe cardiac disease (aOR 2.16, 95% CI 1.08–4.31) were independent predictors for conversion (Table 2). The Hartmann procedure was associated with a higher conversion rate (aOR 2.88, 95% CI 1.35–6.13) than low anterior resections (LAR), with abdominoperineal resections (APR) as reference (Table 2).

**Table 2:** Regression analyses of risk factors for conversion

	Conversion rate (per cent)	Univariable OR (95% CI)	*p* value	Multivariable aOR (95% CI)	*p* value
All patients	95/1284 (7.4)				
Age group					
< 65	37/503 (7.4)	Ref	0.411		
65–80	43/631 (6.8)	0.91 (0.58–4.45)			
> 80	15/150 (10.0)	1.40 (0.75–2.63)			
Sex					
Female	24/502 (4.8)	Ref	0.014	Ref	0.014
Male	71/782 (9.1)	1.98 (1.23–3.21)		1.86 (1.14–3.06)	
WHO ECOG-score					
0, 1	89/1210 (7.4)	Ref	0.974		
2, 3, 4	5/67 (7.5)	1.02 (0.39–2.59)			
ASA classification					
1–2	63/871 (7.2)	Ref	0.742		
3–4	32/413 (7.7)	1.08 (0.69–1.68)			
Severe pulmonary disease					
No	93/1236 (7.5)	Ref	0.391		
Yes	2/48 (4.2)	0.53 (0.29–2.34)			
Severe cardiac disease					
No	83/1211 (6.9)	Ref	0.003	Ref	0.029
Yes	12/73 (16.4)	2.67 (1.39–5.16)		2.16 (1.08–4.31)	
Med. Diabetes					
No	79/1150 (6.9)	Ref	0.036		
Yes	16/134 (11.9)	1.84 (1.04–3.25)			
Weight class (BMI)					
< 18.5	1/24 (4.2)	0.72 (0.09–5.54)	0.007	0.87 (0.12–6.89)	0.002
18.5–25	29/511 (5.7)	Ref		Ref	
25–30	32/496 (6.5)	1.15 (0.68–1.93)		1.08 (0.63–1.83)	
> 30	29/228 (12.7)	2.42 (1.41–4.16)		2.64 (1.51–4.61)	
Radio(chemo)therapy					
No	71/909 (7.8)	Ref	0.381		
Yes	24/375 (6.4)	0.81 (0.50–1.30)			
Operative technique					
LAR	60/743 (8.0)	1.72 (1.03–2.87)	0.012	1.66 (0.97–2.84)	0.021
Hartmann	14/109 (14.3)	2.88 (1.42–5.88)		2.88 (1.35–6.13)	
APR	21/432 (4.8)	Ref		Ref	
Robotic assistance					
No	87/909 (9.6)	Ref	< 0.001	Ref	< 0.001
Yes	8/375 (2.1)	0.21 (0.09–0.43)		0.22 (0.10–0.46)	

A separate analysis on the risk factors gender and BMI revealed an especially high conversion rate for male patients with BMI > 30 in the LR group (Table 3). The OR for conversion in male patients with BMI > 30 was 0.23 (95% CI 0.07–0.83) for RR with LR as reference. A total of 730 patients were included after propensity score matching, with 65 exact matches and 289 fuzzy matches. After propensity score matching, RR compared to LR (aOR 0.19, 95% CI 0.09–0.42) as well as male gender (aOR 2.44, 95% CI 1.14–5.19) remained significant predictors for conversion.

**Table 3:** Rate of conversion stratified by sex and BMI

	Conversion rate		
	RR	LR	OR^a^ (95% CI)
Male (all cases)	6 out of 231 (2.6)	65 out of 551 (11.8)	*p* < 0.001*
Male, BMI > 30	3 out of 52 (5.77)	19 out of 91 (20.88)	0.23 (0.07–0.83) *p* = 0.024
Male, BMI < 30	3 out of 172 (1.74)	42 out of 451 (9.31)	0.17 (0.06–0.57) *p* = 0.004
Female (all cases)	2 out of 114 (1.4)	22 out of 358 (6.1)	*p* = 0.024*
Female, BMI > 30	0 out of 28 (0.00)	7 out of 57 (12.28)	0.12 (0.01–2.15) *p* = 0.149
Female, BMI < 30	2 out of 111 (1.80)	15 out of 297 (5.05)	0.36 (0.08–1.53) *p* = 0.162

Values in parentheses are percentages unless indicated otherwise

*RR* robotic resection, *LR* laparoscopic resection

^a^OR for conversion in RR with LR as reference

*Chi-square analysis

### Postoperative complications

Major complications, 30-day mortality rates and reoperation rates did not differ between the LR and RR group (Table 4). The overall anastomotic leak rate was 41 out of 743 (5.5%) and did not differ between LR and RR. Rates of major complications and reoperations were higher following converted procedures compared to procedures completed laparoscopically, with complication rates of 20 out of 95 (21.1%) vs 135 out of 1189 (11.4%) (*p* = 0.005) and reoperation rates of 13 out of 95 (13.7%) vs 93 out of 1189 (7.8%) (*p* = 0.046).

**Table 4:** Postoperative complications and histopathological results

	LR	RR	*p* values*	CC**	CL***	*p* values*
30-day mortality	3 (0.3)	2 (0.2)	0.592	1 (1.1)	4 (0.3)	0.280
90-day mortality	11 (1.2)	5 (1.3)	0.856	3 (3.2)	13 (1.1)	0.081
Major complications	112 (12.3)	43 (11.5)	0.669	20 (21.2)	135 (11.4)	0.005
Conversion rate	87 (9.6)	8 (2.1)	< 0.001			
Anastomotic leak	27 (4.9)	14 (7.7)	0.203	5 (8.3)	36 (5.3)	0.319
Reoperation	71 (7.8)	35 (9.3)	0.367	13 (12.3)	93 (7.8)	0.046
Tumor perforation	5 (0.6)	2 (0.6)	0.988	3 (3.8)	4 (0.4)	< 0.001
LOS^a^ median (IQR)	6 (4–9)	5 (3–7)	0.001	*8* (6–12)	6 (4–8)	0.001
Single-organ failure	22 (2.4)	5 (1.3)	0.217	3 (3.2)	24 (2.0)	0.456
Multi-organ failure	3 (0.3)	2 (0.5)	0.595	2 (2.1)	3 (0.3)	0.005

Histopathological results						
	LR	RR	*p* values	CC	CL	*p* values
Positive CRM^b^	35 (4.6)	16 (4.8)	0.885	9 (10.2)	42 (4.2)	0.010
Positive DRM^c^	6 (0.8)	1 (0.3)	0.376	1 (1.1)	6 (0.6)	0.547
Median DRM^c^ (IQR)	3.0 (1.8–4.0)	3.5 (2.0–4.5)	0.002	2.6 (2.0–4.0)	3.0 (2.0–4.4)	0.367
Median PRM^d^ (IQR)	15.0 (11.0–20.0)	13.5 (10.0–17.0)	0.001	18.0 (12.0–23.0)	14.0 (10.3–19.0)	0.001
L.node^e^ median (IQR)	16 (12–21)	13 (11–17)	0.001	16 (13–22)	15 (12–20)	0.505
Stage^f^						
1	196 (41.4)	66 (36.1)	0.486	14 (28.6)	248 (40.8)	0.135
2	121 (25.5)	38 (20.8)		11 (22.5)	148 (24.3)	
3	108 (22.8)	54 (29.5)		14 (28.6)	148 (24.3)	
4	49 (10.3)	25 (13.7)		10 (20.4)	64 (10.5)	

Values in parentheses are percentages unless indicated otherwise

*LR* laparoscopic resections, *RR* robotic resections

*Chi-square analyses

**Converted cases

***Completed laparoscopically

^a^LOS, In-hospital length of stay

^b^Circumferential resection margin. Missing values in this variable *n* = 194

^c^Distal resection margin, measured in centimeters. Missing values in this variable *n* = 209

^d^Proximal resection margin, measured in centimeters. Missing values in this variable *n* = 280

^e^Lymph nodes yielded

^f^Missing values in this variable *n* = 627

Conversion, male gender, severe pulmonary or cardiac disease, and BMI > 30 were independent predictors for major complications in multivariable regression analysis (Table 5). After propensity score matching only male gender, severe cardiac disease, and BMI > 30 remained significant. In multivariable regression analysis of 30-day mortality only ECOG-score > 2 was found to be an independent predictor (aOR 21.10, 95% CI 3.27–136.26) *p* = 0.001). For reoperation, male gender (aOR 2.25, 95% CI 1.41–3.59, *p* = 0.001), severe pulmonary disease (aOR 2.74, 95% CI 1.26–5.93, *p* = 0.011), and LAR as operative technique with APR as reference (aOR 2.72, 95% CI 1.64–4.53, *p* < 0.001) were independent predictors in multivariable regression analyses. For anastomotic leak, only male gender was a predictor (aOR 2.44, 95% CI 1.15–5.19, *p* = 0.020). All predictors from initial multivariable logistic regression analysis remained significant in propensity score matched analysis for 30-day mortality rates, reoperations, and anastomotic leak.

**Table 5:** Regression analyses of risk factors for major complications

	Rate (%)	Univariable		Multivariable	
		OR (95% CI)	*p* value	aOR (95% CI)	*p* value
All patients	155/1284 (12.1)				
Age group					
< 65	68/503 (13.5)	Ref	0.192		
65–80	75/631 (11.9)	0.86 (0.61–1.23)			
> 80	12/150 (8.0)	0.56 (0.29–1.06)			
Sex					
Female	43/502 (8.6)	Ref	0.002	Ref	0.009
Male	112/782 (14.3)	1.78 (1.23–2.59)		1.67 (1.14–2.44)	
WHO ECOG-score					
0, 1	148/1210 (12.2)	Ref	0.664		
2, 3, 4	7/67 (10.4)	0.84 (0.38–1.87)			
Severe pulmonary disease					
No	139/1236 (11.2)	Ref	< 0.001	Ref	< 0.001
Yes	16/48 (33.3)	3.95 (2.11–7.48)		3.34 (1.72–6.46)	
Severe cardiac disease					
No	131/1211 (10.8)	Ref	< 0.001	Ref	< 0.001
Yes	24/73 (32.9)	4.04 (2.39–6.79)		3.42 (1.97 (5.94)	
Weight class (BMI)					
< 18.5	2/24 (8.3)	0.98 (0.23–4.35)	0.007		
18.5–25	43/511 (8.4)	Ref			
25–30	70/496 (14.1)	1.79 (1.19–2.67)			
> 30	37/228 (16.2)	2.11 (1.32–3.38)			
Med. Diabetes					
No	135/1150 (10.5)	Ref	0.285		
Yes	20/134 (14.9)	1.32 (0.79–2.19)			
ASA classification					
1–2	102/871 (11.7)	Ref	0.564		
3–4	53/413 (12.8)	1.11 (0.79–1.58)			
Operative technique					
LAR	105/743 (14.1)	Ref	0.020	Ref	0.010
Hartmann	7/109 (6.4)	0.42 (0.19–0.92		0.36 (0.16–0.81)	
APR	43/432 (10.0)	0.67 (0.46–0.78)		0.66 (0.45–0.97)	
Robotic assistance					
No	112/909 (12.3)		0.669		
Yes	43/375 (11.5)	0.92 (0.63–1.34)			
Conversion					
Yes	20/95 (21.1)	2.09 (1.23–5.52)	0.006	1.85 (1.07–3.23)	0.029
No	135/1189 (11.4)	Ref		Ref	

Length of in-hospital stay (LOS) was shorter in the RR group compared to LR; median 5 vs 6 days (*p* = 0.001). Patients who underwent conversion to open access had a median LOS of 8 days compared to 6 days after procedures completed laparoscopically (*p* = 0.001) (Table 4). There were, however, no differences in LOS between LR and RR in hospitals operating with both techniques.

### Histopathological results

The overall rates of positive CRM and DRM were 51 out of 1090 (4.7%) and 7 out of 1075 (0.7%) and were similar in the RR and LR group (Table 4). The rate of positive CRM was higher (9 out of 88, 10.2%) following converted procedures compared to procedures completed laparoscopically (42 out of 1002, 4.2%, *p* = 0.010). A higher proportion of positive CRM was seen following APR compared with other operative techniques (APR 33 out of 357, 9.2%, LAR 12 out of 636, 1.9% and Hartmann 6 out of 97, 6.2%, *p* < 0.001). Further, surgery for low tumors (0–5 cm above anal verge) resulted in higher rates of positive CRM compared with intermediate (5–10 cm) and high (10–15 cm) tumors, with 23 out of 206 (11.2%), 9 out of 297 (3.0%), and 5 out of 250 (2.0%), respectively (*p* < 0.001). Tumor diameter and tumor stage were not associated with higher rates of positive CRM.

A mean number of 14 lymph nodes were retrieved from the specimen in the RR group compared 18 in the LR group (*p* = 0.001). In hospitals performing both LR and RR there were no differences in lymph node retrieval between the two groups, except for one hospital where LR resulted in fewer lymph nodes as compared to RR (Table 6). ANCOVA analysis comparing mean number of lymph nodes between the RR group and the LR group correcting for hospital showed no differences between the two methods (*p* = 0.550).

**Table 6:** Lymph nodes retrieved with LR and RR in hospitals performing both techniques

Center number	*n* total	*n* RR	RR	LR	*p *value
			Mean *n*. lymphnodes (Std.dev)	Mean *n*. lymphnodes (Std.dev)	
1	158	6	26.5 (12.5)	21.1 (11.1)	0.339
2	118	60	13.7 (5.8)	15.1 (4.8)	0.148
3	75	58	15.2 (5.8)	16.1 (5.9)	0.564
4	123	4	20.8 (3.3)	15.7 (7.7)	**0.044**
5	32	19	20.7 (7.8)	26.6 (13.5)	0.174
6	64	34	15.9 (5.9)	16.9 (4.8)	0.482
7	269	194	12.6 (4.9)	13.0 (5.2)	0.562

Significant values (*p* < 0.05) are marked in bold

*RR* robotic resection, *LR* laparoscopic resection

## Discussion

This study on a national cohort of patients who underwent laparoscopic resections for rectal cancer demonstrates that conversion rate was lower with robotic assistance compared to standard laparoscopy. Further, conversion to open access surgery was associated with higher rates of major complications, longer hospital stay, and unfavorable histopathological results.

These results are corroborated by data from a recent meta-analysis of RCTs and propensity score matched studies [17] as well as a large single center study on 600 patients [16], both showing lower conversion rates with robotic assistance compared to conventional laparoscopy in rectal cancer patients, [16, 17]. In contrast, the large international multi-center ROLARR trial found no difference in conversion rates between RR and LR [18]. However, according to a post hoc multi-level logistic regression analysis taking into account the participating surgeon’s experience with robotic surgery, the lack of difference in conversion rates between the two techniques in this multi-center trial could be explained by a learning effect [34].

A conversion rate of 2.1% with RR and 9.6% with LR is generally low compared to other large studies on both laparoscopic and robotic rectal resections, where reported rates vary between 5.0 and 8.1% for RR and 12.2 and 15.4% for LR [16–18]. This could indicate that the operating surgeons had a high level of experience with both robotic assisted and laparoscopic techniques.

Male gender, BMI > 30, and severe cardiac disease were identified as risk factors associated with conversion to open surgery, which is in line with other studies [16, 35, 36]. In a study by Crippa et al., robotic surgery was associated with lower conversion rate in obese patients [14]^.^ In the present study, the conversion rate was especially high for males with BMI > 30 who underwent LR, and the risk for conversion in this group was significantly lower with robotic assistance (Table 3). This indicates that robotic assistance aids in completing surgery laparoscopically especially in the more challenging obese patients combined with a narrow male pelvis. The finding of severe cardiac disease as an independent risk factor for conversion has to our knowledge not been addressed in the literature. The data available for this study do not provide further information to elaborate this finding.

Rates of major complications, 30 day mortality, reoperations, and anastomotic leak did not differ between RR and LR, which is in line with other large studies [7, 16, 18, 37]. While some studies have used standardized complication scores like Accordion grading score [38] or Clavien-Dindo score [5], other studies recorded complications according to custom definitions which vary greatly and make direct comparison difficult. A review of 8 studies including 592 patients undergoing laparoscopic or robotic assisted LAR showed that the overall complication rate was significantly lower in the RR group compared to LR [39]; however, the definition of complications differed between the included studies. In comparison, there were no differences in complication rates between RR and LR in the ROLARR trial comprising 461 patients [18]. The overall rate of major complications in the present study was low, as almost 9 out of 10 patients went through elective rectal cancer surgery without any major complication.

Conversion to open access was followed by higher rates of major complications, reoperations, longer LOS, higher rates of positive CRM, and tumor-near bowel-perforation. Higher rates of complications have been associated with conversion of laparoscopic colon cancer resections in several studies [3–7]. In a study with prospectively collected data of 470 patients who underwent laparoscopic colorectal resections including 192 rectal resections, postoperative complication rates were significantly higher for patients who experienced conversion to open access, with a rate of 56.1% versus 16.8% when resections were completed laparoscopically [37]. This finding is supported by the present study, although the difference in complication rates was less profound.

Histopathological assessment included CRM/DRM and number of retrieved lymph nodes in the specimen. Total number of lymph nodes is one of the key quality measures for assessing the histopathological result following colorectal surgery [40]. The present study showed significantly lower numbers of harvested lymph nodes in the RR group compared with the LR group. However, subgroup analysis indicated that this was related to local hospital or laboratory differences rather than between RR and LR, as there was no difference in number of retrieved lymph nodes after LR and RR in hospitals operating with both methods. Large differences between pathology laboratories in lymph node retrieval have previously been shown in other studies [41, 42]. In the present study, the proportion of patients with neoadjuvant treatment was significantly higher in the RR group. This was probably related to a larger share of low tumors in the RR group which more often meet the criteria for neoadjuvant treatment. Neoadjuvant treatment is well known to be associated with a lower number of specimen lymph nodes. In the ROLARR trial, mean number of lymph nodes retrieved by robotic resections were 24.1, compared to 23.2 for laparoscopic resections [18]. In the COLORII trial the median number of lymph nodes retrieved was 13 for the laparoscopic resections [7], which compares well with the present study.

The overall positive CRM was 4.7% in the present study, which is lower than both the COLORII trial [7] (7.05% for LR) and the ROLARR trial [18] (6.3% for LR and 5.1% for RR). In the present study, positive CRM was more frequent in converted cases, low tumors and tumors resected by APR. Despite a higher proportion of APR and lower tumors in the RR group, no difference was seen regarding positive CRM. This could indicate that robotic assistance reduces the risk for involved CRM in patients operated with APR. In this study however, the reason for conversion was not recorded. In a review [43] of 18 studies on colorectal cancer patients, 3 studies on rectal cancer patients stated that the most common reasons for conversion were advanced tumors, obesity, narrow pelvis, and adhesions. The higher rates of positive CRM in specimens from converted procedures could reflect difficult laparoscopic dissection where conversion to open access enabled to finalize the procedure but could not undo the damage caused by suboptimal dissection.

There are some limitations to this study. The completeness of the mesorectal fascia is an important histopathological quality measure [44, 45], but this variable was not available from the Norwegian Colorectal Cancer Registry. Another limitation is that NoRGast is a newly established registry with low coverage rates during the first years of inclusion. Furthermore, it is possible that surgeons performing robotic rectal resections are those who previously had developed high surgical skills in conventional laparoscopy. However, rectal cancer surgery in Norway has been centralized before the introduction of conventional laparoscopic rectal resection, and the same surgeons are performing LR and RR at centers offering both techniques. The higher conversion rate in LR also in these centers makes this bias unlikely.

Moreover, the present study is an observational study, and the low conversion rate associated with robotic resection could be a result of confounders which were not recorded as variables in the registries. However, separate analyses on hospital level to detect whether conversion rate was dependent on robot system accessibility, showed significantly higher conversion rates with LR also in hospitals with access to such operating systems. Furthermore, propensity score matching was also performed to eliminate bias otherwise only accounted for by an RCT.

This study is based on compound data from two national quality registries covering the surgical and oncological quality of surgical treatment of rectal cancer and shows real time results from treatment outside the strict frames of an RCT. Mandatory inclusion of patients from all hospitals performing rectal cancer surgery enables the possibility to obtain a large dataset of unselected patient population suited for research using advanced statistical methods to minimize bias and confounding. This approach offers results that reflect national daily practice. The degree of external validity would depend on a similar homogenous population and healthcare provision.
